# The Anatomical Breast Burden Model: A Schnur Scale Alternative for Identifying Need for Therapeutic Reduction Mammaplasty

**DOI:** 10.1093/asjof/ojaf168

**Published:** 2025-12-18

**Authors:** Eliana Jolkovsky, Meghan N Miller, Weston McClain, Samantha Rabinovich, Ainaz Dory Barkhordarzadeh, Derrick Lin, Stacy Piva, Addee Lerner, Ginger C Slack

## Abstract

The Schnur sliding scale is used by many insurance companies to determine eligibility for reduction mammaplasty. However, it overlooks anatomical features, symptoms, and physical findings, leading to inequities in coverage determinations. The aim of the study was to develop and validate the anatomical breast burden (ABB) model—a 0-6-point scoring system that quantifies the burden of breasts using breast measurements, physical findings, and symptoms—as an alternative tool to the Schnur scale. A retrospective chart review was conducted for 84 patients who underwent breast reduction at a single academic center. Resection weights, Schnur threshold weights, demographics, and preoperative breast measurements were recorded. ABB cutoff values for sternal notch-to-nipple, nipple-to-inframammary fold, and base width distances were determined statistically, and a score was assigned to each patient. Spearman correlation tested the associations between ABB score and BMI, body surface area, breast measurements, ptosis, resection weight, and the discrepancy between actual resection weight and the Schnur threshold weight. Among patients with an ABB score of 6 (very severe burden), 44% were ineligible for coverage under the Schnur scale, whereas 33% of patients with an ABB score of 2 (mild-to-moderate burden) were eligible. When stratified as low (0-2) and high (3-6) burden, the Schnur scale showed 47.5% sensitivity and 66.7% specificity for identifying patients with significant breast burden. Our model demonstrated stronger correlations with anatomical measurements and resection weight than the Schnur scale. The ABB model better reflects true breast burden and offers a more equitable tool for guiding coverage decisions in breast reduction.

**Level of Evidence**: 3 (Therapeutic) 
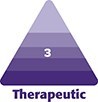

Reduction mammaplasty is one of the most common breast surgical procedures performed in the United States, with more than 76,000 performed in 2023 alone.^1^ Commonly known as breast reduction, it involves removal of breast tissue and skin and transposition of the nipple-areola complex to reduce the size of the breasts. Reduction mammaplasty can be life-changing for patients with macromastia and gigantomastia. Macromastia, a condition of excessively large breast tissue, can cause physical symptoms such as back, neck, and shoulder pain, shoulder grooving because of compression of brassiere straps, intertriginous rashes in the inframammary fold, headaches, and upper extremity paresthesias.^2,3^ Park et al found that, when compared with women with symptomatic macromastia who had not received a breast reduction, women who underwent breast reduction showed significantly lower rates of anxiety, depression, and use of antidepressants.^4^

The current system in the United States for determining insurance coverage for mammaplasty reduction is not uniform. However, most insurers provide coverage to patients based on body habitus and the weight of resected tissue, while ignoring important factors such as symptoms and signs.^5^ In a 2020 cross-section study by Rawes et al of US insurance policies on reduction mammaplasty, 88% (*n* = 42 of 48) of the insurers studied requested a minimum resection volume, with 85% citing the Schnur sliding scale.^6^

Based on a formula that solely relies on a patient's body surface area (BSA), the scale was developed in 1991 to differentiate cosmetic from medically indicated breast reduction.^7^ Reliance on BSA—which is derived from height and weight—to adjudicate whether a reduction would be therapeutic for a patient introduces 2 major limitations. First, the scale inherently discriminates against patients with larger body habitus, as it requires a heavier resection weight for patients with greater BSA even if they have a significant macromastia. Second, the Schnur scale ignores symptoms and physical signs caused by large breasts that may affect a patient's daily life activities. Formulas including the Seitchik, Appel, Galveston, and Descamps models have been cited in literature to estimate resection weight for reduction. The “Seitchik formula” was developed in 1995 from a retrospective insurance study and sought to relate body weight to resection weight. Despite lacking scientific validity, the Schnur and Seitchik models are cited by some insurers.^8^

More recently, Yan et al found that the Schnur scale correlated poorly with actual resection weight (*r*^2^ = 0.38) compared with alternative formulas such as the Appel formula (*r*^2^ = 0.64), Gavelston formula (*r*^2^ = 0.67), and Descamps formula (*r*^2^ = 0.57).^9^ Zhu et al likewise demonstrated that models incorporating direct anatomical breast measurements outperform BSA-based predictors.^10^ However, these models remain mass predictive and ignore important factors such as symptom burden and physical examination findings.

The authors propose an alternative method—the anatomical breast burden (ABB) model—for identifying functional reduction mammaplasty candidates. Rather than relying solely on BSA to determine insurance coverage, we propose a scoring system (0-6) that incorporates breast anatomy, patient symptomatology, and physical examination findings. Our model is not a proposed tool for diagnosis of macromastia or gigantomastia, rather a tool to more accurately quantify the burden that large breasts impose on a patient.

## METHODS

### Patient Enrollment and Data Collection

This retrospective, single-site study was approved by IRB of the UCLA Health System. A report with 299 patients who underwent unilateral or bilateral breast reduction at UCLA Health (Los Angeles, CA) from January 2018 to April 2024 was provided to our study team by the UCLA Clinical and Translational Science Institute (CTSI) from the electronic health record system. The report excluded patients with a gender identity disorder diagnosis or previous implants. The authors conducted chart reviews for 135 patients, reviewing surgeon-documented consult and preoperative notes for breast metrics and pathology reports for the weight of resected breast tissue. Of the 135 patients, 51 did not have documentation of one or more of the following variables: sternal notch-to-nipple distance (SN-N), nipple-to-inframammary fold distance (N-IMF), base width (BW), Regnault ptosis grade, or breast resection weight.

Chart review and data entry were performed incrementally in batches of ∼10 to 15 complete cases to ensure consistent data quality and range representation. Once measurement distributions remained consistent across successive batches, data collection was considered complete, yielding 84 fully documented cases for ABB model derivation and analysis. We extracted the breast metric variables of interest and specimen weights for these 84 patients. If a surgeon documented a ptosis grade of “2-3,” the ptosis grade was recorded in our dataset as 2.5. Demographic information, including height, weight, age, and BMI immediately before surgery, were collected from charts. The breast reductions and corresponding preoperative measurements in this study were performed by 10 different surgeons.

### Schnur Threshold Determination

BSA using the height and weight for each of the 84 patients was calculated in Microsoft Excel (Microsoft Corp., Redmond, WA) using the DuBois formula.^11^ A Schnur threshold weight was assigned to each patient based on their BSA.^12^ To compare each patient's Schnur threshold to the actual weight of resected breast tissue, we computed “mismatch weights” (actual weight − Schnur threshold weight). A positive mismatch indicated that the patient met the Schnur threshold and was thus eligible for insurance coverage. A negative mismatch indicated that the patient would not have met the threshold and therefore would have been considered ineligible.

### Anatomical Breast Burden Model Development

The ABB model, scored zero (no anatomical burden) to 6 (very severe anatomical burden), was designed by our study team. Height, weight, BMI, and BSA were not involved in the development of the model. It comprises 3 domains: (1) patient-reported symptoms, (2) physical findings, and (3) anatomical measurements. The anatomical measurements included in the model are SN-N, N-IMF, BW, and ptosis grade. Each of the 4 anatomical parameters contribute 1 point to the ABB score, whereas the symptom and physical finding domains contribute 1 point each. This weighting structure was designed to emphasize the objective anatomical component of breast burden while still accounting for functional impact through symptoms and physical findings. Additionally, the symptom and physical finding domains include an “Other” category, allowing more flexibility for these domains.

The patient-reported symptoms listed in the ABB scoring rubric include back, neck, and/or shoulder pain, persistent headaches, upper extremity paresthesia, reduced capacity to exercise, sleep disturbances, emotional and psychological distress, and difficulty with daily activities, such as dressing, maintaining hygiene, and lifting objects. These specific symptoms were selected through review of published literature on the most common symptoms reported by patients presenting with macromastia or gigantomastia.^2,4,13-17^ The physical examination findings selected for inclusion in the scoring sheet are inframammary rash, shoulder indentation, anterior shoulder roll, and thoracic kyphosis (Appendix, available online at https://doi.org/10.1093/asjof/ojaf168). These items reflect review of literature of common physical manifestations of breast hypertrophy.^18-22^

The metrics SN-N, N-IMF, and BW were included in our model as they are standard in-office breast measurements documented by surgeons. We incorporated Regnault ptosis grade (0-3), which is similarly reported by surgeons, as ptosis resulting from breast hypertrophy can result in symptoms such as inframammary pruritus. Only severe ptosis (Grade 3) was assigned 1 point in our model to reflect the greatest degree of anatomical burden related to nipple descent. Grades 0 (no ptosis), 1, and 2 were scored as 0, consistent with the ABB model's binary weighting for each anatomical parameter. Altogether, SN-N, N-IMF, BW, and ptosis grade serve to provide a 3-dimensional representation of a breast profile, with SN-N and N-IMF representing the vertical pole, BW representing the horizontal pole, and SN-N and ptosis representing the degree of nipple descent.

The cutoff values for SN-N, N-IMF, and BW were determined statistically in Microsoft Excel using our dataset of 84 patients. Each cutoff was established based on actual resected tissue weight and ptosis grade. To compare cutoff candidates, mean specimen weight and mean ptosis grade were calculated at each integer value of SN-N, N-IMF, and BW in our cohort. For N-IMF and BW, cutoff values that reflect a transition from <500 to ≥500 g, and a transition from moderate-to-severe ptosis (∼2.5) to severe ptosis (∼3), were selected. Because our model already assigns 1 point for severe ptosis, a slightly more lenient cutoff was chosen for SN-N, as it is often used as a surrogate for ptosis. Although these inflection points roughly aligned with the historical 500 g reference weight used by some insurers, the ABB model was intentionally designed to move beyond this policy-based threshold by incorporating anatomical features, physical findings, and symptoms to more accurately reflect true breast burden.^9^

**Figure 1.**
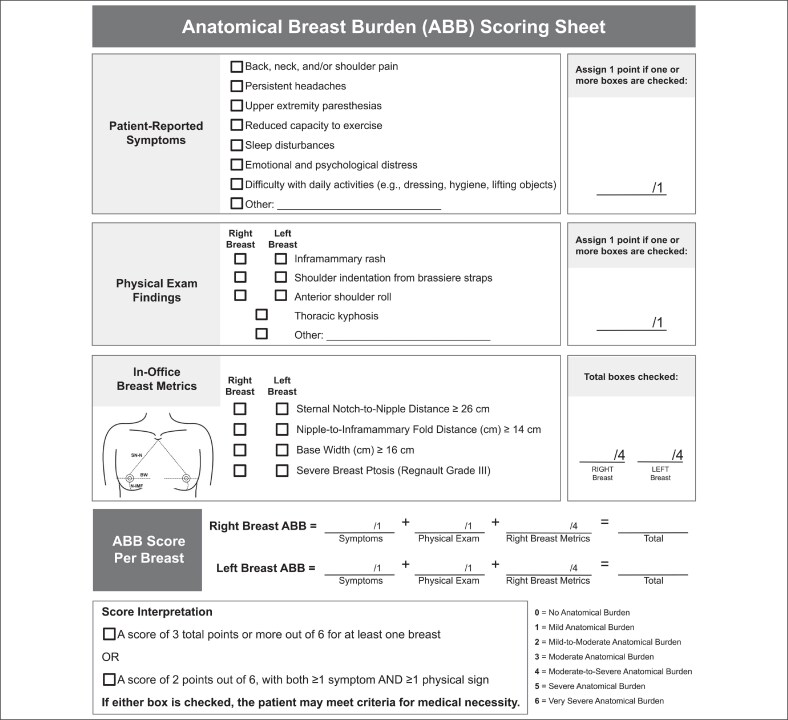
Anatomical breast burden (ABB) scoring rubric.

For SN-N, a cutoff was chosen to reflect a transition of ∼350 to 500 g of weight and a transition in our dataset from moderate (∼2) to moderate-to-severe (∼2.5) ptosis. Although a slightly higher cutoff would have more precisely crossed the 500 g landmark, we selected 26 cm to account for the fact that severe ptosis (3) already contributes 1 point within our model. This slightly more lenient anatomical cutoff avoids overemphasizing nipple descent. Our final ABB scoring rubric is shown in Figure 1.

### Anatomical Breast Burden Score Determination

We analyzed our cohort per patient, rather than per breast, to reflect the health insurance system. For patients who underwent bilateral breast reduction, the breast with the heavier resected weight was chosen for our ABB score determination. We used this system, rather than calculating an average of the 2 specimen weights, because the heavier breast represents a patient's true breast burden and an average weight would underrepresent a patient's true burden. For example, if a patient who underwent bilateral breast reduction had 440 g removed from their right breast and 520 g removed from their left breast, we computed their ABB score using the SN-N, N-IMF, BW, and ptosis grade for their left breast. In this study, we assume that each patient has at least 1 symptom (+1 point) and at least 1 physical finding (+1 point) associated with breast hypertrophy. We then assigned each patient a final ABB score based on their documented breast metrics according to the criteria in Figure 1.

### Statistical Analysis

Statistical analyses were performed in Microsoft Excel. Spearman's correlation coefficients were determined to assess the associations between ABB score and BSA, BMI, breast metrics (ptosis grade, BW, SN-N, and N-IMF), weight of resected breast tissue, and the mismatch between actual resected tissue weight and the Schnur threshold weight. *P*-values for each correlation test were determined to assess the significance of each correlation. Spearman's correlation coefficients and corresponding *P*-values were subsequently calculated for Schnur threshold weights and the same variables for comparison with ABB score. All statistical analyses were 2-tailed. Statistical significance was defined as *P* < .05 (*α* = .05).

## RESULTS

### Demographic Information and Breast Metrics

A total of 84 patients with complete data were included in our final analysis. All patients were female, with a mean age of 49 years (standard deviation [SD] = 16), mean BMI of 28 kg/m^2^ (SD = 3.9), and mean BSA of 1.84 m^2^ (SD = 0.15). The cohort had a mean BW of 15.7 cm (SD = 2.32), mean SN-N of 30.5 cm (SD = 4.04), and a mean N-IMF of 13.8 cm (SD = 2.58). On average, 452 g (SD = 211) of breast tissue was resected per breast (Table 1). All patients exhibited some degree of ptosis. The cohort predominantly demonstrated moderate-to-severe ptosis, with 2% Grade 1 (*n* = 2), 31% Grade 2 (*n* = 26), 10% Grade 2-3 (*n* = 8), and 57% Grade 3 (*n* = 48).

**Table 1.** Patient Demographic Information and Breast Characteristics (*n* = 84)

	Category	Range (minimum-maximum)	Mean	Standard deviation
Patient demographic information	Age (years)	16-86	48.8	15.9
	Height (m)	1.47-1.78	1.63	0.07
	Weight (kg)	53.3-118.8	75.1	11.4
	BMI (kg/m^2^)	18.8-40.4	28.2	3.9
	Body surface area (m^2^)	1.50-2.37	1.84	0.15
Breast characteristics	Regnault ptosis grade (0-3), *n* (%)	1-3	2.6	0.52
	Grade 1 (mild): 2 (2%)	—	—	—
	Grade 2 (moderate): 26 (31%)	—	—	—
	Grade 2-3 (moderate-to-severe): 8 (10%)	—	—	—
	Grade 3 (severe): 48 (57%)	—	—	—
	Sternal notch-to-nipple distance (cm)	22.5-40.0	30.5	4
	Nipple-to-IMF distance (cm)	9-22	13.8	2.6
	Base width (cm)	12-24	15.7	2.3
	Resected breast tissue weight (g) (individual breast)	95-1170	452	211
	Schnur scale threshold weight (g)	260-1167	477	147
	Mismatch weight (actual-Schnur threshold) (g)	−797 to 595	−25	213

Eight patients were recorded as “ptosis grade 2-3” and assigned an interpolated value of 2.5 for analysis to preserve data integrity.

### Schnur Threshold Determination

Table 2 shows the cutoff values for SN-N, N-IMF, and BW, along with the mean ptosis grade and mean breast specimen weights for patients below and above each threshold. For N-IMF, 14 cm was chosen; this distance represented a transition from <500 to >500 g (357-566 g), and a transition from moderate-to-severe to severe ptosis (Grade 2.48-2.74). Following the same logic, a BW of 16 cm or greater was selected to represent 1 point in the ABB model. This value similarly represents the transition from a mean specimen weight of 393 to 534 g, and a mean ptosis grade of 2.45 to 2.80; 26 cm and greater was selected for SN-N. The mean tissue weight for patients with an SN-N of <26 cm was 348, and 467 g for patients ≥26 cm. Although a slightly greater SN-N value represented the true transition across the 500 g landmark weight, 26 cm was selected as severe ptosis (Grade 3) already contributes to 1 of the 6 points in the ABB model.

**Table 2.** Comparison of Breast Metrics Below and Above ABB Cutoffs

Breast measurement	Value	No. of patients (%)	Mean breast tissue weight (g)	Mean ptosis grade (0-3)
Notch-to-nipple distance (cm)	<26	11 (13)	348	2.23
	≥26	73 (87)	467	2.65
Nipple-to-IMF distance (cm)	<14	46 (55)	357	2.48
	≥14	38 (45)	566	2.74
Base width (cm)	<16	49 (58)	393	2.45
	≥16	35 (42)	534	2.80

ABB, anatomical breast burden; IMF, inframammary fold.

### Correlations With Schnur Threshold Weight vs Anatomical Breast Burden Score

**Figure 2.**
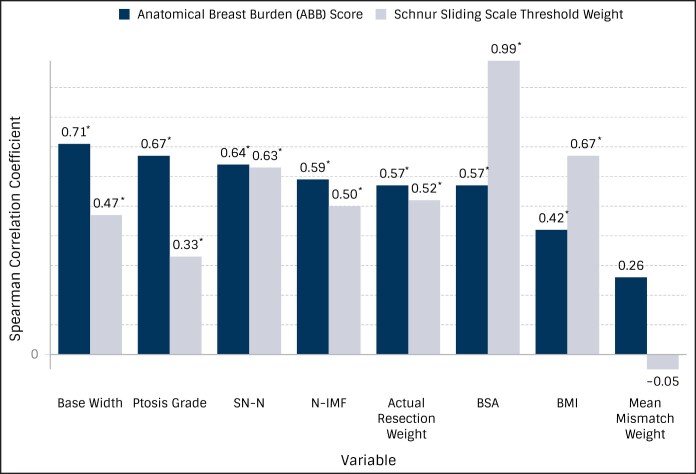
Correlation of anatomical breast burden (ABB) score and Schnur threshold weight with body surface area (BSA), BMI, and breast metrics. Correlations with *P* < .05 are indicated with an asterisk.

Spearman correlation coefficients and corresponding *P*-values for ABB score and Schnur threshold weight with breast metrics, actual specimen weight, BSA, and BMI are shown in Figure 2 and Supplemental Table 1. ABB score had the greatest correlation with BW (*ρ* = 0.71; positive, strong), followed by Regnault ptosis grade (*ρ* = 0.67; positive, strong), SN-N (*ρ* = 0.64; positive, strong), N-IMF (*ρ* = 0.59; positive, moderate), weight of resected breast tissue (*ρ* = 0.57; positive, moderate), BSA (*ρ* = 0.57; positive, moderate), and BMI (*ρ* = 0.42; positive, moderate). As expected, Schnur threshold weight had a correlation with BSA of nearly 1 (*ρ* = 0.99; positive, very strong). BMI was the second strongest correlator with Schnur threshold weight (*ρ* = 0.67; positive, strong), followed by SN-N (*ρ* = 0.63; positive, strong), weight of resected breast tissue (*ρ* = 0.52; positive, moderate), N-IMF (*ρ* = 0.50; positive, moderate), BW (*ρ* = 0.47; positive, moderate), and Regnault ptosis grade (*ρ* = 0.33; positive, weak). All the correlations had significant *P*-values (Supplemental Table 1). Mean mismatch weight (g) demonstrated a weak, yet significant correlation with ABB (*ρ* = 0.26; *P* = .015), and no significant correlation with Schnur threshold weight (g) (*ρ* = −0.05; *P* = .623).

### Distribution of Insurance Coverage Eligibility Across Anatomical Breast Burden and Schnur Scale Criteria

**Figure 3.**
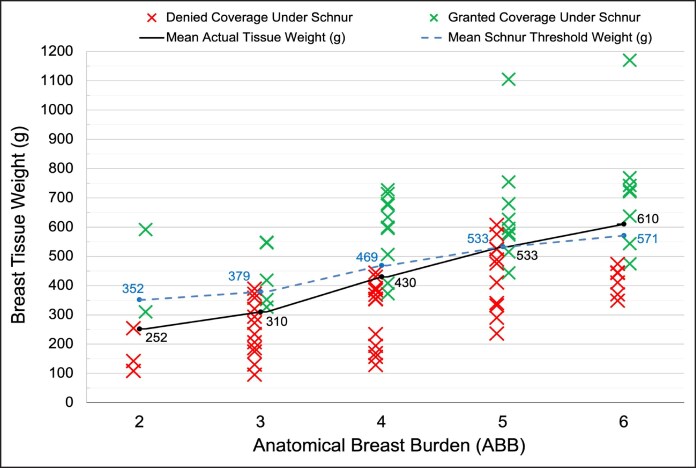
Theoretical insurance coverage outcomes according to the Schnur sliding scale, stratified by anatomical breast burden (ABB) score. These data represent theoretical coverage determinations based on whether patients’ resection weights met or fell below the Schnur threshold.

Table 3 highlights the discrepancies between ABB score and Schnur threshold weights, stratifying patients by whether they would qualify for insurance coverage based on ABB score and the Schnur scale. A total of 61 patients had moderate-to-severe ABB (3-6) according to our model. Of these patients, 29 (47.5%) were eligible for insurance coverage under the Schnur scale (true positives) and 32 (52.5%) were ineligible for coverage despite high breast burden (false negatives). The remaining 24 patients were assigned as mild ABB (0-2). Eight (33%) of these patients met the Schnur threshold weight (false positives) and 16 (67%) did not (true negatives). When stratifying patients as mild ABB and moderate-to-severe ABB, the Schnur scale showed low sensitivity (47.5%) and moderate specificity (66.7%) for identifying patients with significant ABB.

**Table 3.** Stratification of Coverage Eligibility With ABB vs Schnur Scale Across BSA, BMI, and Breast Tissue Weight

		Moderate-to-severe ABB (3-6) (*n* = 61)		Mild ABB (0-2) (*n* = 24)	
		Met Schnur threshold (true positives) (*n* = 29)	Did not meet Schnur threshold (false negatives) (*n* = 32)	Met Schnur threshold (false positives) (*n* = 8)	Did not meet Schnur threshold (true negatives) (*n* = 16)
Body surface area (m^2^)	Mean	1.87	1.91	1.66	1.74
	Minimum	1.59	1.62	1.50	1.55
	Maximum	2.07	2.37	1.82	1.91
	SD	0.11	0.16	0.10	0.11
BMI (kg/m^2^)	Mean	29.5	28.9	24.3	26.4
	Minimum	22.8	23.8	21.6	18.8
	Maximum	37.7	40.4	27.3	34.8
	SD	3.8	3.6	1.9	3.7
Actual resection weight (g)	Mean	657	383	430	228
	Minimum	372	128	310	95
	Maximum	1170	607	591	389
	SD	172	121	114	99
Mean mismatch weight (g)	Mean	+165	−160	+95	−163
	Minimum	+2	−797	+26	−341
	Maximum	+595	−7	+178	−17
	SD	139	180	63	99
Schnur threshold weight (g)	Mean	492	542	335	391
	Minimum	284	310	260	284
	Maximum	687	1167	441	527
	SD	93	182	58	72

ABB, anatomical breast burden; BSA, body surface area; SD, standard deviation.

Among patients with moderate-to-severe breast burden, the mean BSA was similar between patients who met the Schnur threshold and those who did not; 1.87 and 1.91, respectively (Table 3). Similarly, the mean BMI showed no meaningful difference between the 2 groups: 29.5 and 28.9, respectively. The mean BMI and BSA values for patients with mild breast burden were lower than the moderate-to-severe ABB cohort. On average, patients with mild ABB who were eligible for coverage under the Schnur scale had a BSA of 1.66 and a BMI of 24.3. Patients with mild ABB who were ineligible for coverage under the Schnur scale had a mean BSA of 1.74 and BMI of 26.5.

True positives (ABB 3-6; met Schnur threshold) had the highest mean weight of resected breast tissue: 657 g. False negatives (ABB 3-6; did not meet Schnur threshold) had a mean weight of resected tissue of 383 g and a mean mismatch of −160 g below the Schnur threshold. Notably, 1 patient in this group who had an ABB score of 5 out of 6 was ineligible for coverage according to the Schnur scale by merely 7 g. Although this patient had severe ptosis (Grade 3), an SN-N of 29 cm, and a BW of 20 cm, their BSA ultimately disqualified them from coverage.

Table 4 further stratifies patients by individual ABB scores. Notably, 7 of 16 (44%) patients who scored the maximum ABB grade of 6 were ineligible for coverage under the Schnur scale, whereas 33% of patients with an ABB score of 2—who did not meet any anatomical ABB cutoffs—were granted insurance coverage. BSA, BMI, actual resection weight, mean mismatch weight, and Schnur threshold weight all increased in a stepwise fashion with increasing ABB score (Table 4). However, the mean Schnur threshold weight for each ABB score increased at a slower rate than the actual weight of tissue resected; 352 to 571 g and 252 to 610 g, respectively (Figure 3).

**Table 4.** Stratification of BSA, BMI, and Breast Tissue Weight by Individual ABB Score

		ABB score					Schnur sliding scale	
		2 (*n* = 6)	3 (*n* = 18)	4 (*n* = 23)	5 (*n* = 21)	6 (*n* = 16)	Did not meet threshold weight (*n* = 40)	Met threshold weight (*n* = 31)
(m^2^)	Mean	1.68	1.72	1.84	1.91	1.94	1.85	1.83
	Minimum	1.58	1.50	1.59	1.70	1.72	1.55	1.50
	Maximum	1.82	1.91	2.23	2.14	2.37	2.37	2.07
	SD	0.09	0.11	0.13	0.11	0.18	0.16	0.14
ass Index (kg/m^2^)	Mean	24.4	26.2	28.4	29.0	30.4	28.1	28.4
	Minimum	18.8	21.6	23.8	24.8	22.8	18.8	21.6
	Maximum	27.4	34.8	32.4	37.7	40.4	40.4	37.7
	SD	3.2	3.3	2.7	3.2	5.1	3.8	4.1
	Mean	252	310	430	533	610	331	608
	Minimum	108	95	128	236	348	95	310
	Maximum	591	548	727	1105	1170	607	1170
	SD	185	126	184	184	217	135	186
	Mean	−100	−69	−39	+0.47	+33	−161	+150
	Minimum	−262	−341	−451	−285	−797	−797	+2
	Maximum	+150	+178	+273	+530	+595	−7	+595
	SD	165	151	190	181	330	156	129
	Mean	352	379	469	533	571	492	458
	Minimum	284	260	284	370	370	284	260
	Maximum	441	527	895	750	1167	1167	687
	SD	55	77	119	98	216	170	108
Met Schnur threshold weight, *n* (%)		2 (33%)	6 (33%)	10 (43%)	9 (43%)	9 (56%)		
Did not meet Schnur threshold weight, *n* (%)		4 (67%)	12 (67%)	13 (57%)	12 (57%)	7 (44%)		

ABB, anatomical breast burden; BSA, body surface area; SD, standard deviation.

## DISCUSSION

The Schnur scale is based on a 1991 study that correlated the weight of breast tissues removed with the total BSA of the patient.^7^ Before the scale was adopted, most health insurance companies did not provide coverage of reduction mammaplasty.^23^ Many health insurance companies quickly adopted the model to cut costs and draw a clear line between medically indicated reconstructive and aesthetic surgeries.^24^ In the following decades, the Schnur model became an increasingly common industry standard with more than half of 90 insurance companies surveyed using the scale or a modified version of it in 2008.^25^ Despite its commonplace use, the scale is both a poor predictor for patients who would benefit from symptomatic relief, and also of resection weight.^9^ Furthermore, research shows that it systematically discriminates against women based on body habitus.^6^ Insurance companies have denied countless women access to breast reductions. Insurance plans deny breast reductions ostensibly to reduce spending. The existing body of literature, however, shows that macromastia can cause many other health problems such as anxiety; depression; use of antidepressants; back, neck, and shoulder pain; shoulder grooving; intertriginous rashes; headaches; and upper extremity paresthesia. Over the years, researchers have called for insurance companies to create a new standard so that women will be able to undergo this important medical procedure. Notably, Paul Schnur, the author of the original model, has called for a more holistic approach, a conclusion shared by the American Society of Plastic Surgeons.^23,26^

Here, we propose the ABB model with aims to provide a reasonable and structured criterion to effectively differentiate between cosmetic and medically necessary surgeries. The ABB model, which is scored from 0 to 6, quantifies the functional impact of large breasts on a patient. It utilizes commonly recorded breast measurements including sternal SN-N, N-IMF, BW, and Regnault ptosis grade, alongside symptom reports and physical examination findings. It was created on the principle that excessively large breasts can cause physical harm regardless of their proportionality to overall body size. That is, a patient with breasts individually weighing 600 g, for example, can suffer from musculoskeletal burden whether they have a small or large BSA; their back, shoulders, and neck are still burdened with perpetually supporting nearly 3 pounds of breast tissue.

Age may also influence perceived breast burden. Sigurdson et al found that younger patients may report less chronic pain but greater psychological symptoms, whereas physical pain symptoms predominated in the older group.^27^ This variation is accommodated in the ABB model's “Patient-Reported Symptoms” section, which encompasses both physical and psychological components of breast burden.

Our data show the several additional advantages of the ABB model over the Schnur scale. ABB score has a stronger correlation than the Schnur scale with actual weight of resected breast tissue. ABB score showed a stepwise increase in actual resection weight, reflecting the model's ability to stratify patients by true anatomical and symptomatic burden. As breasts become anatomically larger, heavier, and more ptotic, ABB score increases in proportion. In our cohort, 52.5% (*n* = 32 of 61) of patients who were ineligible for coverage under the Schnur scale were assigned a severe or moderate-to-severe breast burden score (Table 3). Many of these patients reported severe symptoms related to their large breasts. Meanwhile, 33% (*n* = 8 of 24) of patients who were assigned a mild breast burden score were granted coverage under Schnur.

The disparity between ABB and Schnur-based coverage determinations highlights the potential inequities created by BSA-dependent criteria. For example, 1 patient with an actual resection weight of 607 g was ineligible for coverage, whereas a patient with 310 g removed was granted coverage. The patient with an actual resected weight of 607 g reported severe back and neck pain related to breast hypertrophy, brassiere strap grooving despite wearing heavy-support brassieres, difficulty with exercise, and excessive inframammary moisture collection and malodor. Before surgical evaluation, the patient attempted symptomatic relief with various nonsurgical therapies such as analgesics. Upon physical evaluation, the surgeon noted severe bilateral breast hypertrophy, Grade 3 ptosis, and moist inframammary folds. This patient, who wore a 36G brassiere on a 5-foot-5-inch frame and endorsed worsening symptoms, missed the Schnur threshold of 628 g by merely 21 g. The ABB model assigns this patient a score of 5 (severe anatomical burden), granting them coverage. Another patient, with Grade 1 ptosis, less severe symptoms, “mild breast hypertrophy,” and half the amount of tissue resected (310 g) was granted coverage under the Schnur scale. This patient scored a 2 (mild anatomical burden) on the ABB scale, not granting them coverage. The disparity between actual clinical burden and Schnur threshold weight are illustrated in Figure 3.

Additionally, our model addresses ptosis severity and breast asymmetry, which is ignored by the Schnur scale. The correlation between ABB score and ptosis grade is twice as strong as that of the Schnur scale with ptosis. Because an increase in ptosis grade results in a deeper inframammary fold and thus increases risk of inframammary moisture collection and intertrigo, it should be a consideration in quantifying a patient's breast burden. Our model acknowledges breast asymmetry by using the breast with greater metrics rather than computing an average of the 2 weights as with the Schnur scale. Averaging the weights of the 2 breasts can minimize and underestimate the real breast burden that a patient with asymmetry experiences. In these patients, the heavier breast contributes disproportionately to their symptoms and physical strain and should be the one considered for insurance coverage decisions.

This study has certain methodological considerations that should be acknowledged when interpreting the findings. Although our study included a diverse group of patients at a large urban hospital, it was limited by a relatively small sample size. However, because we a priori assumed the presence of at least 1 symptom and at least 1 physical examination finding related to breast burden, these domains did not increase covariate load or consume statistical power. Only anatomical variables varied across patients. Because detailed symptom and physical finding data were not consistently available in the retrospective review, the ABB model assumes the presence of at least 1 symptom and 1 physical finding for all included patients. This assumption is consistent with the inclusion of only patients who underwent functional breast reduction for symptomatic macromastia.

A future study would benefit from a larger sample size, other patient populations, and prospective or multicenter data collection to validate the ABB model without a priori assumptions. In addition, future studies could test whether ABB score correlates with pain level, ability to perform activities of daily living, quality of life, or psychological symptoms. The ABB model is not proprietary and was designed as an open, clinically accessible tool. We encourage its further application and external validation at other institutions to confirm reproducibility and generalizability across broader patient populations.

Additionally, we recognize that although our model was designed to estimate a patient's ABB with statistically determined cutoffs, there may be some cases in which a patient does not meet any of the measurement thresholds yet has signs and symptoms and a relatively large amount of tissue resected. For example, 1 patient in our study was assigned an ABB score of 2 (mild breast burden) yet had 591 g resected per breast. These cases may require case-by-case adjudication.

Finally, in 8 instances where ptosis was recorded in chart notes as “Grade 2-3,” an interpolated value of 2.5 was assigned for analysis. Although not part of the formal Regnault system, this approach was used to preserve data integrity and reflect real-world clinical variability in surgeon documentation.

Moving forward, private insurance companies should adopt a more comprehensive model than mere resection weight.^28^ To incentivize private insurance companies to adopt a new scale and expand access to reduction mammaplasty, future researchers could attempt to quantify the cost savings to insurance from prevention of these resultant health conditions. For example, insurance companies may avoid the costs associated with expensive biologic creams for intertriginous rashes by affording women the option of reduction mammaplasty. Legislative and regulatory bodies can also improve coverage of reduction mammaplasty. Advocates for policy change could emphasize both direct health improvements associated with reduction mammaplasty as well as improvements to quality of life and even externalities such as the impact of improved health on relationships and employment.^29^

There are several options to improve coverage. Congress could amend the Affordable Care Act to include breast reduction surgery in the list of Essential Health Benefits.^30,31^ Such legislation could encourage adoption of a symptomatic-focused scale. Alternatively, the Department of Health and Human Services may issue regulatory guidance requiring insurance companies to use the ABB model. Congress could alternatively pass a more focused law like the Women's Health and Cancer Rights Act of 1998 (WHCRA) which requires insurance companies to cover postmastectomy reconstruction.^32^ Further, the federal government could explore covering reduction mammaplasty through Medicare or the Veteran's Administration before implementing a mandate on private insurance companies. Finally, advocates may pursue change on a state-by-state basis. This may be a more realistic method given Washington's partisan gridlock in recent decades. State legislatures could enact mandates requiring medically indicated breast reduction surgeries, and regulatory bodies may interpret statutes to promote use of the ABB model or similarly holistic approaches.

## CONCLUSIONS

We propose an alternative model to the Schnur sliding scale, which is commonly used by insurance companies to determine coverage for reduction mammaplasty. The 0- to 6-point ABB model quantifies the functional burden imposed on a patient by large breasts using standard anatomical measurements, patient-reported symptoms, and physical examination findings. Grounded on the principle that large breasts—regardless of proportionality to the body frame—can inflict physical and functional harm on a patient, the ABB model demonstrated stronger correlations with resection weight and clinical breast features than the Schnur scale. Our findings support its potential as a more equitable and anatomically grounded tool for assessing medical necessity in breast reduction.

## Supplementary Material

ojaf168_Supplementary_Data
