# Supplementary material for: The Anatomical Breast Burden Model: A Schnur Scale Alternative for Identifying Need for Therapeutic Reduction Mammaplasty
Source: Aesthet Surg J Open Forum. 2025 Dec 18;8:ojaf168. doi: 10.1093/asjof/ojaf168 (PMC12853872; doi:10.1093/asjof/ojaf168)
Supplement: ojaf168_Supplementary_Data [file ojaf168_supplementary_data.zip › Appendix.pdf]

Anatomical Breast Burden (ABB) Scoring Sheet

Patient-Reported Symptoms

- ☐ Back, neck, and/or shoulder pain
- ☐ Persistent headaches
- ☐ Upper extremity paresthesias
- ☐ Reduced capacity to exercise
- ☐ Sleep disturbances
- ☐ Emotional and psychological distress
- ☐ Difficulty with daily activities (e.g., dressing, hygiene, lifting objects)
- ☐ Other: ______________________________

Assign 1 point if one or more boxes are checked:

_______ /1

Physical Exam Findings

Right Breast	Left Breast	
☐	☐	Inframammary rash
☐	☐	Shoulder indentation from brassiere straps
☐	☐	Anterior shoulder roll
☐		Thoracic kyphosis
☐		Other: ______________________________

Assign 1 point if one or more boxes are checked:

_______ /1

In-Office Breast Metrics

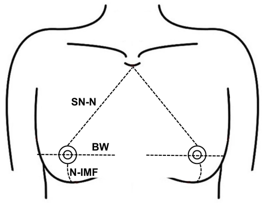

Right Breast	Left Breast	
☐	☐	Sternal Notch-to-Nipple Distance ≥ 26 cm
☐	☐	Nipple-to-Inframammary Fold Distance (cm) ≥ 14 cm
☐	☐	Base Width (cm) ≥ 16 cm
☐	☐	Severe Breast Ptosis (Regnault Grade III)

Total boxes checked:

_______ /4 RIGHT Breast   _______ /4 LEFT Breast

ABB Score Per Breast

Right Breast ABB = _______ /1 (Symptoms) + _______ /1 (Physical Exam) + _______ /4 (Right Breast Metrics) = _______ (Total)

Left Breast ABB = _______ /1 (Symptoms) + _______ /1 (Physical Exam) + _______ /4 (Right Breast Metrics) = _______ (Total)

Score Interpretation

☐ A score of 3 total points or more out of 6 for at least one breast

OR

☐ A score of 2 points out of 6, with both ≥1 symptom AND ≥1 physical sign

If either box is checked, the patient may meet criteria for medical necessity.

- **0** = No Anatomical Burden
- **1** = Mild Anatomical Burden
- **2** = Mild-to-Moderate Anatomical Burden
- **3** = Moderate Anatomical Burden
- **4** = Moderate-to-Severe Anatomical Burden
- **5** = Severe Anatomical Burden
- **6** = Very Severe Anatomical Burden
