# Supplementary material for: The Anatomical Breast Burden Model: A Schnur Scale Alternative for Identifying Need for Therapeutic Reduction Mammaplasty
Source: Aesthet Surg J Open Forum. 2025 Dec 18;8:ojaf168. doi: 10.1093/asjof/ojaf168 (PMC12853872; doi:10.1093/asjof/ojaf168)
Supplement: ojaf168_Supplementary_Data [file ojaf168_supplementary_data.zip › Supplemental Table 1.docx]

**Supplemental Table 1.** Correlation of ABB Score and Schnur Threshold Weight with BSA, BMI and Breast Metrics

|  | Anatomical Breast Burden (ABB)  (n = 84) | | Schnur Sliding Scale Threshold Weight  (n = 84) | |
| --- | --- | --- | --- | --- |
|  | Spearman correlation coefficient, ρ | P-value | Spearman correlation coefficient, ρ | P-value |
| Base Width (cm) | 0.71 | < 0.001 | 0.47 | < 0.001 |
| Breast Ptosis Grade | 0.67 | < 0.001 | 0.33 | 0.002 |
| SN-to-Nipple Distance (cm) | 0.64 | < 0.001 | 0.63 | < 0.001 |
| Nipple-to-IMF Distance (cm) | 0.59 | < 0.001 | 0.50 | < 0.001 |
| Actual Resection Weight (g) | 0.57 | < 0.001 | 0.52 | < 0.001 |
| Body Surface Area (m^2^) | 0.57 | < 0.001 | 0.99 | < 0.001 |
| Body Mass Index (kg/m^2^) | 0.42 | < 0.001 | 0.67 | < 0.001 |
| Mean Mismatch Weight (g) | 0.26 | 0.015 | –0.05 | 0.623 |

Strongest correlations for ABB and Schnur are bolded.
